# Moderating effect of coping strategies on the association between perceived discrimination and blood pressure outcomes among young Black mothers in the InterGEN study

**DOI:** 10.3934/publichealth.2025014

**Published:** 2025-02-17

**Authors:** Alexandria Nyembwe, Yihong Zhao, Billy A. Caceres, Kelli Hall, Laura Prescott, Stephanie Potts-Thompson, Morgan T. Morrison, Cindy Crusto, Jacquelyn Y. Taylor

**Affiliations:** 1 Columbia University School of Nursing, Center for Research on People of Color, New York, NY, USA; 2 Columbia University School of Nursing, New York, NY, USA; 3 Tulane University School of Public Health and Tropical Medicine, New Orleans, USA; 4 Yale University School of Medicine, 333 Cedar Street, New Haven, CT, USA

**Keywords:** discrimination, coping, blood pressure, Black women, Black mothers

## Abstract

Research suggests experiences of racial discrimination influence blood pressure outcomes among Black women, but little is known about how coping strategies may influence this relationship. Our study aimed to assess the moderating effects of coping strategies on perceived racial discrimination and blood pressure among young Black mothers. We conducted a secondary analysis on data from the Intergenerational Impact of Genetic and Psychological Factors on Blood Pressure study. Eligible participants were African American or Black women aged 21 and older, who did not present with any cognitive disorder that may obscure reporting data, and who had a biological child who was 3–5 years old at the time of study enrollment. In our analysis, systolic and diastolic blood pressure were the primary outcomes, and experiences of discrimination situations and frequency subscales were the primary predictors. We considered the three subscales of the Coping Strategy Indicator (problem-solving, seeking social support, and avoidance) as moderators. Linear regression models were used. Of the 246 female participants (mean age: 31.3 years; SD = 5.8), the mean systolic and diastolic blood pressures were 114 mmHg (SD = 13.8) and 73 mmHg (SD = 10.9), respectively. The frequency of experiences of perceived racial discrimination was significantly associated with higher systolic blood pressure, but this relationship was moderated among participants with greater seeking social support scores (p = 0.01). There were no significant moderation effects in models with diastolic blood pressure as the outcome. Future studies should examine this relationship longitudinally and further investigate specific coping strategies Black women use to manage perceived racial discrimination.

## 1. Introduction

Hypertension among Black adults, compared to other racial/ethnic groups, has an earlier onset [1] and greater acceleration from pre-hypertension to hypertension [2]. Research demonstrates that lifestyle modifications may improve blood pressure; however, environmental factors, including experiences of perceived discrimination, may also have a profound effect. There is a growing body of literature demonstrating that experiences of discrimination among Black adults are associated with an increased risk of heart disease [3]–[5], including hypertension risk [6]–[9]. These findings are especially concerning among Black women; although mortality rates related to cardiovascular disease (CVD) have declined [10],[11], compared to White women, Black women have higher mortality rates [12]. One plausible explanation for this higher risk is the link between chronic stressors, such as discrimination, and CVD [13].

Racial discrimination affects various minority groups across the United States. Many Black Americans migrated to the North to achieve economic stability and escape the discrimination prevalent in the South. However, discrimination persisted in the North as well, through practices like redlining and restrictive real estate practices, which sought to confine Black people to specific neighborhoods. As the population of Black Americans in the North grew, racial residential segregation intensified, which had long-term effects on economic and social opportunities for Black people [14]. Black women, specifically, report experiences with racial discrimination throughout their lives beginning in childhood with their own experiences and extending into adulthood, with Black mothers additionally facing the chronic stress of witnessing their children's experiences with racial discrimination [15]. Unmanaged, chronic mental stress can have significant effects on the health and well-being of Black women [16], including increased hypertension risk [17],[18]. Based on these findings, it becomes necessary to also bolster coping strategies among Black women, particularly Black mothers.

Coping relates to the mental and behavioral strategies people use to manage, lessen, or endure the challenges and pressures brought on by stressful situations [19]. Black women, in general, reportedly use multiple techniques to manage the varying degrees of stress experienced in their day-to-day lives, including prayer and other spiritual practices [20],[21], and seeking social support [22]. Black mothers may employ additional coping strategies to manage daily stressors related to parenting, including seeking social support specifically in neighborhood relationships [23]. Regardless of the coping mechanism used, evidence suggests that Black women may feel obligated to balance multiple stressors while also upholding the narrative that Black women are strong and must push through adversity while helping others, despite the amount of stress they may face [24].

While literature examining experiences of racial discrimination and its influence on blood pressure outcomes among Black mothers continues to grow, little is known about the moderating effect of coping strategies on the relationship between experiences of perceived racial discrimination and blood pressure in young Black mothers. To address this question, we examined these relationships using data from a longitudinal study of Black women and their young children to examine the effects of genetic and environmental stressors on these mother-child dyads. We hypothesize that 1) a greater number of experiences of perceived racial discrimination will be positively associated with systolic and diastolic blood pressure, and 2) coping strategies will moderate the relationship between experiences of perceived racial discrimination and systolic and diastolic blood pressure.

## 2. Materials and methods

### 2.1. Methods

We conducted a secondary data analysis to examine whether coping strategies moderated the associations of experiences of racial discrimination and blood pressure among Black mothers in the Intergenerational Impact of Genetic and Psychological Factors on Blood Pressure (InterGEN) study. The details of this study are explained elsewhere [25],[26]. Briefly, a sample of 250 mother–child pairs was recruited between 2014 and 2019 from socioeconomically disadvantaged, ethnically and racially diverse urban communities through collaborations with early care and education centers in Connecticut offering preschool education. This longitudinal cohort examined the interaction of genes and environmental factors on blood pressure in both mothers and their children. Recruitment sites were provided with study materials (e.g., brochures and flyers) for distribution to potential families. Potential participants were approached by study staff during tabling events to inform them of the study aims and, if interested, later contacted for a telephone screening to ensure eligibility. Participants were eligible to participate in the InterGEN study if they 1) were at least 21 years of age, 2) self-identified as African American or Black, 3) spoke English, 4) did not present with any cognitive disorder that could obscure reporting data, and 5) were enrolled with a biological child who was 3–5 years old at the time of study enrollment. Written informed consent was obtained from all interested study participants. Participants were interviewed at four time points: T1 (baseline), T2 (6 months), T3 (12 months), and T4 (18 months). Data that support the findings of this study are available from the final author upon reasonable request.

#### 2.1.1. Samples

For this study, we used data from the T1 collection point only. Of the 250 mothers who participated in the parent study, 246 had complete data on all variables for the present analysis, so our sample size was 246. We used clinical data [including blood pressure, height, weight, and body mass index (BMI)] and psychosocial data (including experiences of racial discrimination and coping strategies). All psychosocial data were collected using the audio computer-assisted self-interviewing (ACASI) method to aid in minimizing in-person interviewer bias. Data collection occurred at a convenient location of the participants' choice such as their home, a local library, or their child's early childhood care center. IRB approval for this study was obtained from Yale University (#1311012986).

### 2.2. Measures

#### 2.2.1. Primary outcome variable: blood pressure

Trained personnel manually collected blood pressure three times in the left arm of participants who were seated. The Joint National Committee-7 guidelines [27] were followed. The systolic and diastolic blood pressure values were averaged across the three readings for each participant to create one systolic and diastolic blood pressure outcome per person.

#### 2.2.2. Primary exposure variable: discrimination

Our primary exposure variable was the mother's self-reported racial discrimination experience, which was measured by the Experiences of Discrimination (EOD) Scale [28]. The EOD measures self-reported experiences of racial discrimination in adults of all races/ethnicities from working backgrounds. We used the EOD discrimination subscale, which includes the Situation and Frequency versions. The Situation version is a nine-item questionnaire measuring specific situations in which a person may experience discrimination because of their race, ethnicity, or color. Example settings include work, school, getting service in a store or restaurant, and obtaining credit, bank loans, or a mortgage. Scores range from 0 to 9. The Frequency version assessed how many times each of the nine discriminatory experiences occurred due to race or ethnicity. The following values were assigned: 0 to never, 1 to once, 2.5 to 2–3 times, and 5 to 4 or more times. These values were summed across all nine items. Scores range from 0 to 45.

#### 2.2.3. Moderating variable: coping

The 33-item Coping Strategy Indicator (CSI) measures coping strategies in response to one stressful event within the last six months [29]. The CSI includes three subscales: problem-solving, seeking social support, and avoidance coping. This CSI and its subscales have been previously validated among a sample of African Americans [30]. Items are scored using a Likert scale of 1 (not at all) to 3 (a lot). Scores for each subscale range from 11 to 33, with higher scores indicating greater use of each strategy. In this sample, Cronbach's alphas for problem-solving and seeking social support were between 0.93 and 0.94, while that for the avoidance subscale was 0.83.

#### 2.2.4. Covariates

To mitigate potential confounding effects, we adjusted all models for a set of covariates including age, race (Black vs. Mixed), education level (high school diploma/GED or less, some college or associate degree, college degree or above), annual household income (household in which the child lives the majority of the year), employment status in the past 12 months, marital status, smoking history, and BMI (kg/m^2^). These covariates were included in the models due to their established associations with blood pressure [31]–[35].

#### 2.2.5. Statistical analysis

Continuous variables are summarized by means and standard deviations, while categorical variables are reported in terms of frequencies and percentages. In our primary analyses, we aimed to assess the significant effects of the interaction between each discrimination variable and each coping variable on the outcome measures. For each combination of outcome and exposure variables, we built three separate multiple linear regression models that included a single moderating variable. All covariates listed above were controlled in each model. All statistical tests were two-sided. To address the issue of multiple comparisons, we controlled the test significance using the false discovery rate approach, setting the rate at 0.05. For the models that revealed a significant interaction effect, an interaction plot was generated to illustrate how coping modified the relationship between discrimination and the outcome variables. All statistical analyses were carried out using R version 4.3.0 [36].

## 3. Results

Sample characteristics are described in Table 1. The sample consisted of 246 female participants, with a mean age of 31.3 years (SD = 5.8). More than one-third (36.2%) had a high school diploma or GED, while 33.3% had some college education but no degree, and 14.2% had a bachelor's degree or above. Two-thirds (66.7%) of the participants were employed at the time of the study, but more than half (52.4%) of participants made under $20,000 per year in income. More than half (64.6%) of the sample was single, and less than one-quarter (22.0%) were current smokers. Mean systolic and diastolic blood pressures were 114 mmHg (SD = 13.8) and 73 mmHg (SD = 10.9), respectively. On average, participants reported at least one type of situation in which perceived racial discrimination was experienced. The most frequently reported situations in which experiences of racial discrimination occurred included when getting service from a store or restaurant (24.0%), getting hired for a job (23.17%), and while on the street or in a public setting (21.54%).

**Table 1.** Sample characteristics.

	Overall (N = 246)
**Age** mean (SD)	31.3 (5.8)
**Race** n (%)	
Black	230 (93.5%)
Mixed	16 (6.5%)
**Education** n (%)	
Less than high school	13 (5.3%)
High school diploma or GED	89 (36.2%)
Some college, no degree	82 (33.3%)
Associate's degree	27 (11.0%)
Bachelor's, Master's, or Doctorate degree	35 (14.2%)
**Marital Status** n (%)	
Married	59 (24%)
Single	159 (64.6%)
Divorced	12 (4.9%)
Separated	3 (1.2%)
Living with but not married to significant other	13 (5.3%)
**Employment** n (%)	
Employed	164 (66.7%)
Unemployed	77 (31.3%)
Missing	5 (2.0%)
**Annual Income** n (%)	
Less than $5000	54 (22.0%)
$5000–$9999	30 (12.2%)
$10,000–$14,999	26 (10.6%)
$15,000–$19,999	19 (7.7%)
$20,000–$24,999	22 (8.9%)
$25,000–$34,999	31 (12.6%)
$35,000–$49,999	30 (12.2%)
$50,000 or higher	25 (10.2%)
Missing	9 (3.7%)
**BMI** mean (SD)	29.8 (8.4)
**Smoking** n (%)	
No	191 (77.6%)
Yes	54 (22.0%)
Missing	1 (0.4%)
**Systolic blood pressure** mean (SD)	114 (13.8)
**Diastolic blood pressure** mean (SD)	72.6 (10.9)
**Experiences of Discrimination Subscales** mean (SD)	
Situation scores	1.45 (1.9)
Frequency scores	3.67 (6.0)
**Coping Strategy Indicator** mean (SD)	
Seeking social support	21.7 (7.3)
Problem solving	25.4 (7.6)
Avoidance	18.7 (5.9)
Total score	65.8 (17.7)

### 3.1. Moderation analysis results with systolic blood pressure as the outcome

**Figure 1.**
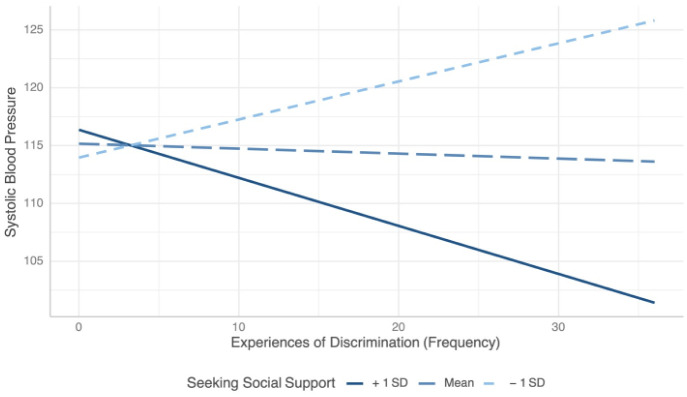
Interaction effects of EOD Frequency score and seeking social support on systolic blood pressure. Lines represent the relationship between EOD Frequency score and systolic blood pressure at three seeking social support levels: +1 SD (above mean), mean, and −1 SD (below mean).

When systolic blood pressure was treated as the outcome in the model, our regression analyses showed that EOD frequency scores were significantly associated with higher systolic blood pressure (b = 1.06, p = 0.02) (Table 2). However, this relationship was moderated and inversed among those with higher levels of seeking social support, as evidenced by the significant interaction term (EOD frequency × seeking social support: b = −0.05, p = 0.01). Figure 1 illustrates how seeking social support moderated the EOD–systolic blood pressure relationship. Specifically, our model showed a positive association between EOD frequency and systolic blood pressure among those with low levels of seeking social support. Among individuals with higher levels of social support, there was an inverse association between experiences of discrimination (EOD) and systolic blood pressure. Also, in our adjusted analyses, neither problem-solving (b = −0.00, p = 0.88) nor avoidance coping (b = −0.04, p = 0.45) significantly moderated the association between perceived racial discrimination frequency and systolic blood pressure (Table 3). Similarly, interaction terms including EOD Situation and seeking social support (b = −0.14, p = 0.13), problem-solving (b = −0.02, p = 1.00), and avoidance coping (b = −0.13, p = 0.37) were linked to lower systolic blood pressure, but these results did not reach statistical significance (Table 4).

**Table 2.** Regression results for assessing the moderation effect of seeking social support on the relationship between EOD frequency score and systolic blood pressure.

**Variable**	**Estimates (95% CI)**	**p-value**
(Intercept)	77.16 (63.24–91.09)	<0.001
EOD Frequency scores	1.06 (0.16–1.97)	**0.021**
Seeking social support	0.17 (–0.10–0.44)	0.228
Race (Mixed)	−0.37 (−7.03–6.28)	0.912
Age	0.61 (0.31–0.92)	<0.001
Income	−0.66 (−1.48–0.17)	0.119
Education (Ref: High School/GED)		
Bachelor's, Master's, or Doctorate degree	3.54 (−2.44–9.51)	0.245
Associate's degree	2.92 (−3.06–8.90)	0.337
Some college, no degree	0.35 (−3.68–4.37)	0.866
Less than high school	6.81 (−0.83–14.44)	0.081
Employment status (Yes)	−0.22 (−3.96–3.51)	0.907
BMI	0.61 (0.41–0.80)	<0.001
Smoking (Yes)	3.26 (−0.68–7.20)	0.105
Marital status (Ref: Married)		
Single	−3.84 (−8.12–0.44)	0.078
Other	−3.59 (−9.52–2.34)	0.234
EOD Frequency scores × seeking social support	−0.05 (−0.09−0.01)	**0.006**

Note: *Moderation model adjusted for age, race (Black vs. Mixed), education level, annual household income, employment status in the past 12 months, BMI, marital status, and smoking history; +EOD, Experiences of Discrimination; BMI, body mass index.

### 3.2. Moderation analysis results with diastolic blood pressure as the outcome

As planned, we performed extensive moderation analyses to assess whether any of the coping subscales moderated the relationships between the two primary discrimination variables (EOD Situation and EOD Frequency) and diastolic blood pressure outcomes. After multiple comparison adjustments, no significant moderation effects were observed in models with diastolic blood pressure as the outcome. In our adjusted analyses, interaction terms including EOD Frequency and seeking social support (b = −0.03, p = 0.59), problem-solving (b = −0.01, p = 0.63), and avoidance coping (b = −0.02, p = 0.62) were associated with lower diastolic blood pressure but did not reach statistical significance (Table 3). Similarly, interactions terms including EOD Situation and seeking social support (b = −0.09, p = 0.42), problem-solving (b = −0.06, p = 1.00), and avoidance coping (b = −0.11, p = 0.42) were associated with lower diastolic blood pressure but did not reach statistical significance (Table 4).

**Table 3.** Moderating effect of coping on maternal experiences of discrimination–Frequency and blood pressure.

Predictor	Outcome	Estimate	Std error	T-statistics	P-value	Adjusted P-value
EOD_Frequency	SYSTOLIC BP	1.06	0.46	2.32	0.02	
CSI_SSS	SYSTOLIC BP	0.17	0.14	1.21	0.23	
EOD_Frequency: CSI_SSS	SYSTOLIC BP	−0.05	0.02	−2.77	0.01	0.04*
EOD_Frequency	SYSTOLIC BP	−0.04	0.75	−0.05	0.96	
CSI_PROB	SYSTOLIC BP	−0.02	0.14	−0.17	0.86	
EOD_Frequency: CSI_PROB	SYSTOLIC BP	−0.00	0.02	−0.15	0.88	0.88
EOD_Frequency	SYSTOLIC BP	0.58	0.47	1.25	0.21	
CSI_AVOID	SYSTOLIC BP	0.18	0.18	1.01	0.31	
EOD_Frequency: CSI_AVOID	SYSTOLIC BP	−0.04	0.02	−1.66	0.10	0.45
EOD_Frequency	DIASTOLIC BP	0.49	0.38	1.28	0.20	
CSI_SSS	DIASTOLIC BP	0.07	0.11	0.65	0.52	
EOD_Frequency: CSI_SSS	DIASTOLIC BP	−0.03	0.02	−1.73	0.08	0.59
EOD_Frequency	DIASTOLIC BP	0.17	0.62	0.27	0.79	
CSI_PROB	DIASTOLIC BP	−0.09	0.11	−0.82	0.41	
EOD_Frequency: CSI_PROB	DIASTOLIC BP	−0.01	0.02	−0.48	0.63	0.63
EOD_Frequency	DIASTOLIC BP	0.24	0.39	0.62	0.54	
CSI_AVOID	DIASTOLIC BP	0.01	0.15	0.05	0.96	
EOD_Frequency: CSI_AVOID	DIASTOLIC BP	−0.02	0.02	−1.02	0.31	0.62

Note: *p < 0.05; ^a^adjusted for race, age, income, education, employment, BMI, marital status, and smoking history; ^b^EOD_Frequency, Experiences of Discrimination-Frequency; CSI, coping strategy indicator; SSS, seeking social support; CSI_PROB, problem-solving; CSI_AVOID, avoidance coping.

**Table 4.** Moderating effect of coping on maternal experiences of discrimination–Situation and blood pressure.

Predictor	Outcome	Estimate	Std error	T-statistics	P-value	Adjusted P-value
EOD_Situation	SYSTOLIC BP	2.81	1.43	1.96	0.05	
CSI_SSS	SYSTOLIC BP	0.18	0.15	1.21	0.23	
EOD_Situation: CSI_SSS	SYSTOLIC BP	−0.14	0.06	−2.36	0.02	0.13
EOD_Situation	SYSTOLIC BP	0.12	1.95	0.06	0.95	
CSI_PROB	SYSTOLIC BP	−0.02	0.15	−0.11	0.91	
EOD_Situation: CSI_PROB	SYSTOLIC BP	−0.02	0.07	−0.27	0.78	1.00
EOD_Situation	SYSTOLIC BP	2.17	1.51	1.44	0.15	
CSI_AVOID	SYSTOLIC BP	0.24	0.20	1.22	0.22	
EOD_Situation: CSI_AVOID	SYSTOLIC BP	−0.13	0.07	−1.80	0.07	0.37
EOD_Situation	DIASTOLIC BP	1.66	1.19	1.40	0.16	
CSI_SSS	DIASTOLIC BP	0.10	0.12	0.85	0.39	
EOD_Situation CSI_SSS	DIASTOLIC BP	−0.09	0.05	−1.82	0.07	0.42
EOD_Situation	DIASTOLIC BP	1.18	1.61	0.74	0.46	
CSI_PROB	DIASTOLIC BP	−0.06	0.12	−0.46	0.65	
EOD_Situation: CSI_PROB	DIASTOLIC BP	−0.06	0.06	−0.99	0.33	1.00
EOD_Situation	DIASTOLIC BP	1.79	1.24	1.44	0.15	
CSI_AVOID	DIASTOLIC BP	0.11	0.16	0.66	0.51	
EOD_Situation: CSI_AVOID	DIASTOLIC BP	−0.11	0.06	−1.82	0.07	0.42

Note: *p < 0.05; ^a^adjusted for race, age, income, education, employment, BMI, marital status, and smoking history; ^b^EOD_Situation, Experiences of Discrimination-Situation; CSI, coping strategy indicator; SSS, seeking social support; CSI_PROB, problem-solving; CSI_AVOID, avoidance coping.

## 4. Discussion

To expand on existing research that has examined the associations between experiences of racial discrimination and blood pressure outcomes among young Black women, we investigated whether experiences of perceived racial discrimination and blood pressure were moderated by coping strategies among a sample of young adult Black mothers. Our study found that a greater frequency of experiences of racial discrimination was associated with higher systolic blood pressure. Among individuals with higher levels of social support, there was an inverse association between the frequency of experiences of discrimination and systolic blood pressure. Individuals with moderate levels of support experienced a more neutral or less impactful interaction between the frequency of experiences of discrimination and systolic blood pressure. Due to the cross-sectional design of this study, we were unable to establish a causal relationship between perceived racial discrimination and elevated systolic blood pressure. However, it is possible that individuals in our sample with strong social support may experience a buffering effect that mitigates the physiological impact of perceived racial discrimination. We did not find any significant association between experiences of perceived racial discrimination and diastolic blood pressure, nor did we find any significant moderation of coping on this association. Perhaps, moderating effects of coping on the relationship between other forms of discrimination (e.g., age, gender, sexual orientation) and diastolic blood pressure may be significant among young Black women and should be explored further in future studies.

Our findings expand on the existing literature examining whether seeking social support buffers the relationship between stressors related to perceived discrimination and blood pressure among Black populations [37]. Literature suggests that functional (e.g., perceived or tangible) and structural (e.g., marital status, number of and frequency of contact with social connections) social support may act as protective factors that can mitigate stress responses via various physiological pathways [38]. Black women may seek social support from friends, family, and spouses to manage general stress or discrimination as well as racial discrimination [39],[40]. Additionally, Black women may seek to communicate their thoughts or feelings around experiencing discrimination in perceived safe spaces, specifically among other Black adults who may understand their feelings [41]; this may lower hypertension risk over time [42]. Our findings from the current study suggest two possible explanations: 1) seeking social support could be a tool used to buffer the negative impact of perceived racial discrimination on blood pressure, or 2) unmeasured confounding variables may account for these findings. Seeking social support may partially alleviate the physical toll of racial discrimination experiences; however, a longitudinal analysis may be needed to better understand its role in the relationship between perceived racial discrimination and blood pressure. Additionally, we accounted for age, education, income, and BMI, factors known to influence blood pressure, but we did not include other potential confounders, such as duration and quality of sleep [43], physical activity [44], and alcohol consumption [45]. Considering these limitations, our findings highlight the need for further research on this complex relationship between perceived racial discrimination and blood pressure to better understand the association with social support.

While scores for the situation and frequency of experiences of discrimination were relatively low among this sample, the most frequently reported experiences of discrimination from this sample included when getting service from a store or restaurant, when getting hired for a job, and when on the street or in a public setting. These results are similar to those of a telephone survey that included non-Hispanic Black (n = 802) and non-Hispanic White U.S. adults (n = 902) [46]. Bleich et al. [46] found that more than half (57%) of Black adults reported experiencing discrimination in the employment domain because of race. Other literature has found Black women to have reported discrimination experiences in similar domains while pregnant, including in healthcare settings [47],[48], which may influence both whether mothers seek follow-up care postpartum [49] and health outcomes [47]. Institutional and individual-level racism and discrimination may be significant sources of stress for Black Americans. To potentially mitigate the impact of perceived discrimination and other social determinants on cardiovascular health among young Black mothers, healthcare systems could explore partnerships with community-based resources (e.g., community clinics and medical/social outreach programs) [50]. Such efforts have the potential to address the multifaceted stressors among Black mothers and improve the effective management of chronic medical conditions like hypertension. Additionally, future research should further explore the specific situations in which young Black mothers experience perceived racial discrimination during significant time periods such as during pregnancy and postpartum.

The Seeking Social Support subscale from the CSI scale used in this study includes questions about accepting sympathy or understanding from someone, talking to others about a situation to feel better, and seeking reassurance from those who know them best. The most endorsed scale items from our study's participants included 1) talking to people about the situation, because talking about it helped them to feel better, accepting sympathy and understanding from someone, and accepting help from a friend or relative. These endorsements suggest that Black women may seek and accept support from people they trust to hear their concerns and receive empathy. In addition to these coping techniques, Black women may utilize additional strategies to cope with stress. Among Black women, in general, research suggests that religious and spiritual practices as well as resistance techniques such as vocalization are used to cope with various stressful situations, including when experiencing racism and discrimination [41]. The use of each of these strategies could vary based on age, socioeconomic status, personal beliefs, or availability of resources, among other factors. Future studies should strive to better understand the specific reasons for relying on such strategies to cope and explore additional coping styles among Black women, particularly among young Black mothers, and how these coping styles influence blood pressure over time.

### 4.1. Future research directions

In addition to the previously mentioned suggestions for future research, we would like to discuss additional opportunities to address perceived discrimination experienced within the healthcare system by Black women. There is a need for discrimination tools that can further probe into the different forms of discrimination Black women may face. The Experiences of Discrimination scale, for example, does highlight discrimination experienced in various domains, but a subscale asking additional questions about the results of these discrimination experiences that Black women face is needed. Black women report gender discrimination at higher rates in multiple domains (e.g., healthcare, housing, court systems) compared to their White counterparts [51]. Qualitative interviews with Black women and their experiences with discrimination have revealed initial responses to discrimination experiences including active vs. passive coping techniques [15], but future studies could include interviews to further address these coping techniques, willingness to access and utilize active forms of coping, and whether healthcare and other social and institutional resources are sought following these perceived experiences with racial discrimination.

### 4.2. Limitations

While this research adds to the existing literature examining the complex relationship between experiences of perceived discrimination and blood pressure outcomes among Black women, this study has several limitations. First, our sample size was comprised only of young Black mothers within a specific region of the United States. Whether our findings can be generalized for young Black mothers in other geographical locations warrants further research. Second, we did not assess additional variables for coping used among Black women, such as religiosity/spirituality, neighborhood support, or resistance, which could influence the relationship between perceived discrimination experiences and blood pressure. Third, we did not analyze data regarding the sample's children, which may provide further context on factors influencing maternal blood pressure. Fourth, in using the EOD scale, we did not analyze data regarding experiences of discrimination related to gender, sexual orientation, or disability status. Additionally, the EOD measure does not provide a cumulative score for the impact of discrimination across multiple domains, which may enhance the tool. Fifth, our study was cross-sectional; measures such as income, smoking status, education, and coping strategies may change over time. Future research exploring these associations and the moderating effect of coping strategies should be examined over time. Future research should also investigate the intersectionality of stress arising from multiple forms of discrimination and their impact on blood pressure outcomes among Black mothers.

## 5. Conclusion

Blood pressure control remains a concern among minoritized populations, especially Black women. Previous studies have demonstrated a relationship between discrimination experiences and blood pressure among Black adults. In our cross-sectional analysis using a cohort of young Black mothers, seeking social support moderated the relationship between the frequency of experiences of discrimination and systolic blood pressure. Future studies should examine this relationship longitudinally and investigate specific coping strategies Black women may use to manage varying forms of discrimination.

## Use of AI tools declaration

The authors declare they have not used Artificial Intelligence (AI) tools in the creation of this article.
